# How general semen quality influences the blastocyst formation rate: Analysis of 4205 IVF cycles

**DOI:** 10.5935/1518-0557.20180022

**Published:** 2018

**Authors:** Mariana M Piccolomini, Tatiana CS Bonetti, Eduardo La Motta, Paulo C Serafini, Jose R Alegretti

**Affiliations:** 1 Huntington - Medicina Reprodutiva. São Paulo, Brazil; 2 Disciplina de Ginecologia Endocrinológica, Departamento de Ginecologia, Escola Paulista de Medicina da Universidade Federal de São Paulo (UNIFESP-EPM), São Paulo, Brazil; 3 Disciplina de Ginecologia, Departamento de Obstetrícia e Ginecologia, Faculdade de Medicina, Universidade de São Paulo (FMUSP). São Paulo, Brazil

**Keywords:** blastocyst formation rate, semen quality, embryo transfer, *in vitro* fertilization

## Abstract

**Objective:**

To select embryos with higher implantation potential, the extended culture has been the most frequently applied strategy worldwide, and consequently leads to higher live birth rates per transfer. Sperm quality is a determining feature, and it may influence the outcomes of IVF from fertilization to embryo development. Therefore, we hypothesize that blastocyst formation may also be impaired by general semen quality.

**Methods:**

We analyzed 4205 IVF cycles. Four study groups were designed according to semen quality: normal, mild alteration, severe alteration and epididymis. All cycles were intended to extend embryo culture until the blastocyst stage, and embryo development was evaluated.

**Results:**

Regarding cleavage rate, the normal and mild alteration semen groups were equivalent, and the severe alteration and epididymis semen groups were equivalent to each other. The blastocyst formation rate decreased with semen quality. At least one blastocyst formed in 79.9% of cycles for the normal semen group, whereas the percentage of cycles with the formation of at least one blastocyst was slightly lower for the mild alteration (75.6%), severe alteration (76.4%) and epididymis (76.8%) semen groups. A multivariate logistic regression showed that for each additional cleaved embryo on day 3, the chance of having at least one blastocyst doubles. Additionally, the chance of having at least one blastocyst decreased when semen presented mild or severe alterations.

**Conclusion:**

The general quality of sperm is a good predictor of blastocyst formation, significantly affecting the likelihood of having at least one blastocyst at the end of the cycle. Based on our findings, it is necessary to consider general semen quality and the number of cleaved embryos when forecasting the possibility of blastocyst formation and transfer in an extended culture system.

## INTRODUCTION

Extended embryo culture and transfer at the blastocyst stage is an alternative process that enables embryo selection at more advanced stages of development, increasing pregnancy rates and minimizing the risk of multiple pregnancies (Kupka *et al*., 2014). Extended culture has been the most frequently applied strategy worldwide, especially since recent guidelines have emphasized the single embryo transfer approach (Maheshwari *et al*., 2016). Other advantages of extended embryo culture to the blastocyst stage are the possibility of trophectoderm biopsy for genetic analysis, and the time-lapse approach for evaluating embryo development (Zheng *et al*., 2016).

It is clear that blastocyst transfer leads to higher live birth rates per transfer. However, there is a risk of losing embryos that do not survive until day 5 (D5), which ultimately results in lower cumulative live birth rates per couple (Maheshwari *et al*., 2016). The following factors may affect the potential for an embryo to develop to the blastocyst stage: advanced maternal age (Yan *et al*., 2012), paternal age (Dain *et al*., 2011), endometriosis (Borges *et al*., 2015), diminished ovarian reserve (Katz-Jaffe *et al*., 2013) and abnormal sperm quality (Chapuis *et al*., 2017).

Despite the multifactorial characteristics of infertility, sperm quality is a determining feature. The male factor is present in approximately 50% of the cases, regardless of female factors. Previous studies have shown that sperm motility reduction is a critical parameter that affects fertilization rates, number of embryos developed (Chapuis *et al*., 2017) and rate of good quality embryos on day 3 (Zheng *et al*., 2016). Additionally, sperm morphology has been associated with top quality embryo rates at the cleavage stage (day 3) (Meng *et al*., 2016).

Sperm quality is a determining feature which may influence IVF outcomes, from fertilization to embryo development; therefore, we hypothesize that the blastocyst formation rate may also be impaired. This perception is an important aspect of forecasting blastocyst formation rates. Therefore, the aim of this study was to retrospectively evaluate the blastocyst formation rate of different sperm quality groups in a large cohort of IVF cycles.

## MATERIALS AND METHODS

This was a retrospective cohort study involving 4,205 IVF cycles performed between January 2015 and December 2016 at a private reproductive medicine center in Brazil. The study included all consecutive couples with an indication for IVF, submitted to ovarian stimulation with their own oocytes and ejaculate or epididymis sperm. The cycles using testicular sperm were excluded from the study. According to ethical guidelines, institutional review board approval was not required for this study due to its retrospective nature and anonymized data.

### Study design

Ejaculated semen samples were collected by masturbation after 3 to 5 days of ejaculation abstinence. Epididymis sperm samples were collected by epididymis puncture. The samples were analyzed according to World Health Organization (WHO) recommendations, and sperm quality was considered normal for samples with more than 15 million motile spermatozoa, without motility or morphological alterations. Sperm quality was considered abnormal for samples with less than 15 million motile spermatozoa and/or some kind of motility or morphological alteration according to WHO parameters (World Health Organization, 2010). The following four groups were classified according to semen quality, as per WHO (World Health Organization, 2010) criteria:

- Normal: cycles in which the ejaculated semen analysis resulted in normal parameters for concentration, motility and morphology (n=977).
- Mild alteration: cycles in which the ejaculated semen analysis resulted in one or two abnormal parameters for concentration (5-14 million/ml), motility (<6 million/ml) and/or morphology (<4%) (n=2358).
- Severe alteration: cycles in which the ejaculated semen analysis resulted in <5 million sperm/mL or alterations in the three parameters for concentration, motility and morphology (n=724).
- Epididymis: cycles in which epididymis sperm was used (n=146).

### Sperm Preparation

Fresh or cryopreserved semen samples were used for IVF. Epididymis sperm samples were placed in the culture medium (HTF modified, Irvine, USA), supplemented with a 15% synthetic serum substitute (SSS, Irvine Scientific) right after puncture, and washed by centrifugation at 1,600 rpm for 10 min. Both ejaculated and epididymis sample preparations were performed using a medium culture gradient (Isolate, sperm separation medium, Irvine Scientific, USA) according to manufacturer instructions, and were suspended in 0.5 mL of sperm rinse (Vitrolife), and then used for ICSI.

### Ovarian stimulation, oocyte fertilization and embryo culture

All women received controlled ovarian stimulation, according to our clinic's routine protocols. Briefly, pituitary blockade was achieved with a GnRH antagonist (Orgalutran^Ⓡ^ 0.25 mg, MSD) or agonist (Lupron^Ⓡ^, Abbott) according to a standard protocol. Ovarian stimulation was performed with recombinant FSH (Gonal F^Ⓡ^, Merck Serono or Puregon^Ⓡ^, MSD) with and without hMG (Menopur^Ⓡ^, Ferring) and initiated on day 2 or 3 of the menstrual cycle. The initial gonadotrophin dose was determined by the clinical profile of the patient and adjusted according to the ovarian response. Follicle development was monitored by ultrasonographic assessment; when women had at least two follicles that were ≥18 mm in diameter, final oocyte maturation was triggered with 250µg of recombinant hCG (rhCG, Ovidrel^Ⓡ^, Merck Serono). Oocyte aspiration was performed 35-36 hours after triggering.

The oocytes were denuded and then assessed for maturity stage. All mature metaphase II (MII) oocytes were fertilized by intracytoplasmic sperm injection (ICSI) (Palermo *et al*., 1992). On day 1 (D1), the normally fertilized oocytes - defined as having two pronuclei (2PN) and two polar bodies - were identified and cultured in groups until day 3 (D3) in 1 mL of cell culture medium (G-1 Plus, Vitrolife) under a layer of paraffin oil (OVOIL, Vitrolife), in incubators with 5% O_2_ and 5% CO_2_.

From D3 until the blastocyst stage (D5 or D6), the embryos were cultured in 1 mL of medium containing 10% human albumin (CSCM, Irvine Scientific) under a layer of paraffin oil. The embryos were then incubated in triple gas incubators (90% N_2_, 5% O_2_ and 5% CO_2_). The blastocysts were morphologically classified according to Gardner *et al*. (2000). All cycles were intended for extended embryo culture until the blastocyst stage. The cycles in which the embryo did not develop until the blastocyst stage were cancelled. Of the 4,205 cycles, 233 were cancelled due to non-cleaved embryos on D3 (5.5%); from the 3,972 remaining cycles, 744 were cancelled, because the embryos did not develop until the blastocyst stage (18.7%). The cycles with blastocyst formation underwent fresh transfer or blastocyst cryopreservation.

### Data analysis

The primary goal of this study was to determine the blastocyst formation rate, which was calculated by the number of blastocysts per number of fertilized oocytes. The fertilization rate (number of normal fertilized oocytes per number of oocytes injected) and the cleavage embryo rate (number of cleaved embryos per number of normal fertilized oocytes) was also calculated. The results were analyzed based on the four pre-established groups.

The patients' demographic data was evaluated using descriptive statistics and presented as means and frequencies. Continuous variables were compared using mean and frequency comparison tests (ANOVA or Student's t-test and Pearson' X^2^, respectively). Regression analyses were used to evaluate the association between variables. Data analyses were performed using the SPSS 22 (IBM SPSS Software, USA), and we considered *p*-values ≤0.05 to be statistically significant .

## RESULTS

From 4,205 cycles, 32,031 MII oocytes were recovered and 10,925 blastocysts developed. The demographic data of the women included in this study and semen characteristics according to groups are described in Table 1. Despite significant differences found regarding the women's ages, the number of oocytes, MII collected and the numerical values were very close and not clinically relevant. On the other hand, the differences found in the semen analysis were expected due to group classifications.

**Table 1:** Demographic characteristics of women included in the study, and ovarian stimulation outcomes according to study groups

Groups	Normal	Mild alterations	Severe alteration	Epididymis	*p*
Women age (years) (mean±SD)	37.1±4.1	37.3±3.9	36.0±4.0	36.6±4.3	<0.001
Number of oocytes collected (mean±SD)	10.0±7.0	9.8±6.7	11.2±7.0	12.0±8.4	<0.001
Number of MII (mean±SD)	7.60±5.7	7.4±5.4	8.2±5.5	8.9±6.7	<0.001
Semen analysis					
-Concentration	80.0±48.1	52.8±40.3	4.6±3.9	----	<0.001
-Motility	51.1±33.7	31.2±27.2	1.7±1.7	----	<0.001
-Morphology	4.8±1.2	1.9±0.8	1.3±0.6	----	<0.001

**Figure 1:**
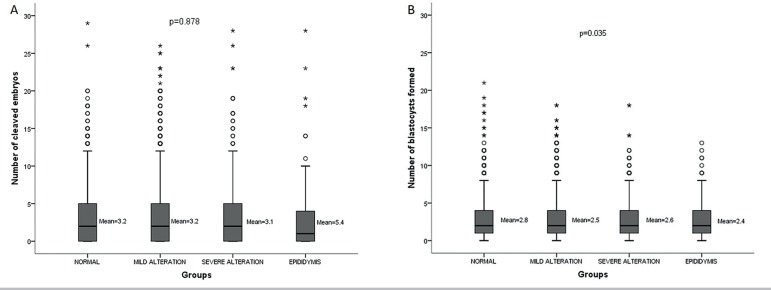
Comparison of number of cleaved embryos on D3 (A) and number of blastocyst formed on D5 (B) in the study groups.

The fertilization rates on study groups were 80.1% for the normal ones, 79.4% for those with mild alteration, 75.4% for those with severe alterations and 70.7% for the epididymis ones (*p<*0.001). Despite the statistical significance concerning fertilization rates, the numerical differences were not clinically important, since all groups had at least 70% of oocytes fertilized after ICSI. The same was found for the number of cleaved embryos and blastocysts formed (Figures 1A and 1B).

**Figure 2:**
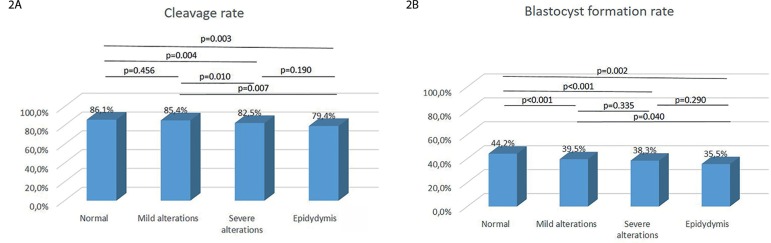
Comparison of (A) cleavage rate (number of cleaved embryos on D3 / number of fertilized) and (B) blastocyst formation rate (number of blastocysts formed on D5 / number of fertilized) in the study groups.

Regarding embryo cleavage rates, the statistical differences on the severe alteration and epididymis groups to the mild alteration and normal groups are probably due to the huge number of cycles included in this study, as the numbers are very similar, and differences are not clinically important (Figure 2A). The same was found for blastocyst formation rates, in which statistical differences do not represent clinical significance (Figure 2B). Additionally, at least one blastocyst formed in 79.9% of the cycles, when the normal semen was used for ICSI - which was significantly lower for the mild alteration group (75.6%, *p*=0.006). But it was not significant when compared to the severe alteration (76.4%, *p*=0.079) and epididymis (76.8% *p*=0.374) semen groups. However, as it happened before, there was no clinical significance concerning the differences.

Aiming to rule-out confounding factors, we built a logistic regression model, to evaluate the influence of semen quality on the likelihood of having at least one blastocyst at D5, adjusted for maternal age, number of MII oocytes collected and embryos cleavage on D3. The adjusted multivariate logistic regression showed that the likelihood of having at least one blastocyst decreased by approximately 50% (*p<*0.001, OR=0.667) and 35% (*p*=0.027, OR=0.738) when semen presented mild alterations and severe alterations, respectively. There was no significant influence of the epididymis sperm group on the likelihood of having at least one blastocyst, despite OR indicating approximately 35% less possibility, which was similar to the semen group with severe alteration (Table 2).

**Table 2:** Multivariate logistic regression model to determine possibility of having at least one blastocyst formed, adjusted for confounders.

	Coefficient	Standard error of Coefficient	*p*value	OR
Women age (years)	-0.084	0.012	<0.001	0.919
Number of MII oocytes recovered	-0.122	0.024	<0.001	0.885
Number of embryos at cleavage stage (D3)	0.846	0.042	<0.001	2.330
Semen with mild alteration	-0.405	0.108	<0.001	0.667
Semen with severe alteration	-0.303	0.137	0.027	0.738
Epididymis sperm	-0.302	0.230	0.188	0.739

## DISCUSSION

Many studies show contradictory results, and there is no consensus as to which seminal parameter (i.e., concentration, motility or morphology) is best for evaluating sperm potential in IVF. Several authors suggest that severe oligospermia is an important factor, it reduces fertilization potential and embryo quality (Meng *et al*., 2016); however, other authors have shown that severe oligospermia has no influence (Chen *et al*., 2009). Moreover, the first proposal of sperm morphology as a predictor of IVF outcomes was by Kruger *et al*. (1986) in the 80s, who reported a relationship between men with an increased proportion of sperm with abnormal morphology and decreased likelihood of pregnancy. Many studies were subsequently carried out, and the Kruger morphology criteria has been considered the main parameter of IVF indication; Kruger's classification is still used as strict criteria in the manual for semen examination of the WHO (World Health Organization, 2010). Morphology defects can hide a genetic abnormal condition of the sperm cells (Magli *et al*., 2012). However, the effect of morphology on the likelihoods of embryo implantation and pregnancy is still contradictory in the literature (De Vos *et al*., 2003; Loutradi *et al*., 2006). Additionally, several authors have recently demonstrated that men presenting 0% of normal sperm are still able to obtain natural pregnancy (Kovac *et al*., 2017). There is a high correlation of sperm motility with the capacity of sperm to reach the oocyte in a natural conception; thus, sperm selection techniques are currently used, pushing the sperm to a motility challenge (swim-up) or forcing them through a differential gradient, aiming to mimic the natural selection characteristics seen in vivo (Sakkas *et al*., 2015).

However, men commonly present not just one alteration, but a combination of sperm defects, and it is necessary to consider the three factors together. In our study, we classified semen samples into four groups, considering all parameters; the presence of three normal parameters was considered the normal group, and three abnormal parameters or a concentration lower than 5 million sperm/ml was considered a severe alteration. Other levels of alteration in one or two semen parameters were considered mild alterations. Epididymis sperm was considered in an individual group. This approach allowed for a broad view of the semen quality and male reproductive potential.

We considered the hypothesis that in ICSI cycles where one spermatozoa with better quality parameters is chosen and injected in the oocyte, the intrinsic quality is still affected by the general semen quality, and it is impossible to tell the best spermatozoa based on the genetic information alone. Therefore, the potential of embryo development is also affected (Zheng *et al*., 2016). Based on the group classifications, we evaluated the effects of semen quality on blastocyst development in a large cohort of patients/oocytes.

Our findings showed that the lower the semen quality, the lower the blastocyst formation rate. However, despite the statistically significant decrease in blastocyst formation, the clinical relevance is small as the difference between the higher (normal semen group=44.2%) and lower (epididymis group=35.5%) blastocyst formation rates is less than 10%. Also, the difference between the mean number of blastocysts formed is only 0.4 (normal semen group=2.8 and epididymis group=2.4).

Studies published more than 2 decades ago report that both diminished sperm morphology quality and concentration lower the likelihood of good morphology embryo formation. However, those semen parameters were evaluated separately, and the embryos were classified based on cleavage stage (Parinaud *et al*., 1993). We also noticed the cleavage rate, and the differences follow the same pattern that blastocysts have; as there is a statistical difference but it is not clinically relevant.

Zheng *et al.* (2016) demonstrated that a reduced number of motile spermatozoa diminished fertility and embryo quality on day 3; however, if there was a good embryo for transfer, the likelihoods of implantation and pregnancy were similar. Then, it was suggested that the implantation rate was the important parameter to evaluate the ability of an individual embryo to be implanted and it was not associated with sperm quality (Zheng *et al*., 2016). We did not evaluate the clinical outcomes, which is a limitation of this study. However, our primary goal was to evaluate the blastocyst formation rate, which is a parameter of embryo quality and implantation potential. There was a greater likelihood of implantation when the embryo was transferred in the blastocyst stage (Alves da Motta *et al*., 1998; Glujovsky *et al*., 2012; Harton *et al*., 2013; Kolibianakis *et al*., 2002; Maheshwari *et al*., 2016).

The blastocyst formation is also dependent on many other factors, and to analyze whether the association of semen quality and the blastocyst formation was independent of oocyte/female factors, we built a multiple logistic regression model adjusted for maternal age, number of MII oocytes recovered and number of cleaved embryos. Considering that normal semen does not influence the presence or absence of one formed blastocyst (dependent variable), patients classified as mild alteration or severe alteration had a significantly lower likelihood of having a blastocyst (50% and 35% less chance, respectively), independent of oocyte/female factors.

We did not find a significant association of epididymis sperm with blastocyst formation, which may be due to a smaller number of cycles included in this group compared to the other groups. However, the effects of epididymal sperm on IVF outcomes is still controversial in the literature (Aboulghar *et al*., 1997; Meniru *et al*., 1998; Nicopoullos *et al*., 2004).

Notably, the logistic regression model also showed that the number of cleaved embryos is a significant predictor of having a blastocyst at the end of the cycle. Our results suggest that poor semen quality decreases the chance of having a blastocyst, despite of univariate analysis had shown a numerically similar blastocyst formation rates. Accordingly, the worse the semen quality, the more cleaved embryos are required for blastocyst formation. Future studies should be performed to establish the better method for embryo transfer, considering both semen quality and number of cleaved embryos.

In summary, we suggest that the general sperm quality, considering the three main parameters of concentration, motility and morphology, can predict the blastocyst formation rate. Hence, it is essential to consider the general semen quality and number of cleaved embryos in counselling couples undergoing IVF with extended culture to blastocyst transfer.
